# Evaluating the efficacy, safety and evolution of renal function with early initiation of everolimus-facilitated tacrolimus reduction in *de novo* liver transplant recipients: Study protocol for a randomized controlled trial

**DOI:** 10.1186/s13063-015-0626-0

**Published:** 2015-03-26

**Authors:** Bjorn Nashan, Peter Schemmer, Felix Braun, Markus Dworak, Peter Wimmer, Hans Schlitt

**Affiliations:** Department of Hepatobiliary Surgery and Visceral Transplantation, University Medical Center Hamburg-Eppendorf, Hamburg, Germany; Department of General, Visceral and Transplant Surgery, University Hospital Heidelberg, Heidelberg, Germany; Department of General Surgery and Thoracic Surgery, University Hospital Schleswig, Kiel, Holstein Germany; Novartis Pharma GmbH, Nürnberg, Germany; Department of Surgery, University Hospital Regensburg, Regensburg, Germany

**Keywords:** Everolimus, Early initiation, Liver transplantation, HCC, MELD

## Abstract

**Background:**

Introduction of calcineurin inhibitors had led to improved survival rates in liver transplant recipients. However, long-term use of calcineurin inhibitors is associated with a higher risk of chronic renal failure, neurotoxicity, *de novo* malignancies, recurrence of hepatitis C viral (HCV) infection and hepatocellular carcinoma. Several studies have shown that everolimus has the potential to provide protection against viral replication, malignancy, and progression of fibrosis, as well as preventing nephrotoxicity by facilitating calcineurin inhibitor reduction without compromising efficacy. The Hephaistos study evaluates the beneficial effects of early initiation of everolimus in *de novo* liver transplant recipients.

**Methods/Design:**

Hephaistos is an ongoing 12-month, multi-center, open-label, controlled study aiming to enroll 330 *de novo* liver transplant recipients from 15 centers across Germany. Patients are randomized in a 1:1 ratio (7–21 days post-transplantation) to receive everolimus (trough levels 3–8 ng/mL) with reduced tacrolimus (trough levels <5 ng/mL), or standard tacrolimus (trough levels 6–10 ng/mL) after entering a run-in period (3–5 days post-transplantation). In the run-in period, patients are treated with induction therapy, mycophenolate mofetil, tacrolimus, and corticosteroids according to local practice. Randomization is stratified by HCV status and model of end-stage liver disease scores at transplantation. The primary objective of the study is to exhibit superior renal function (estimated glomerular filtration rate assessed by the Modification of Diet in Renal Disease (MDRD)-4 formula) with everolimus plus reduced tacrolimus compared to standard tacrolimus at Month 12. Other objectives are: to assess the incidence of treated biopsy-proven acute rejection, graft loss, or death; the incidences of components of the composite efficacy endpoint; renal function via estimated glomerular filtration rate using various formulae (MDRD-4, Nankivell, Cockcroft-Gault, chronic kidney disease epidemiology collaboration and Hoek formulae); the incidence of proteinuria; the incidence of adverse events and serious adverse events; the incidence and severity of cytomegalovirus and HCV infections and HCV-related fibrosis.

**Discussion:**

This study aims to demonstrate superior renal function, comparable efficacy, and safety in *de novo* liver transplant recipients receiving everolimus with reduced tacrolimus compared with standard tacrolimus. This study also evaluates the antiviral benefit by early initiation of everolimus.

**Trial registration:**

NCT01551212.

**Electronic supplementary material:**

The online version of this article (doi:10.1186/s13063-015-0626-0) contains supplementary material, which is available to authorized users.

## Background

### Liver transplantation: challenges for long-term outcomes

Liver transplantation is the most preferred and well-established mode of treatment in patients with end-stage liver diseases. From 1968 to 2009, approximately 93,634 liver transplantations were carried out in 83,816 patients in Europe. Of these, 14,116 liver transplantations were performed in Germany [1] from across 24 centers [2]. Around 1,097 liver transplantations were performed in 2012 and the 1-year patient survival rate is 76.6% (733 of the 957 patients with known survival status) [3]. Analysis of data across centers in Germany (2007 to 2010) reveals that better 1-year overall survival rates were significantly correlated with larger center size (R^2^ = 0.09, *P* = 0.009), whereas in-house mortality (R^2^ = 0.007, *P* = 0.52) and 3-year survival rates (R^2^ = 0.05, *P* = 0.09) showed non-significant correlation between center volume and outcome [4]. Model of end-stage liver disease (MELD) score was introduced in Germany in 2006 for thorough and transparent liver allograft allocation. Following the introduction of MELD, mortality on the waiting list decreased; however, 1-year survival rates also decreased from 90% to less than 80% [5,6]. The match MELD threshold increased from 25 to 34 [6,7]. A vast number (~40%) of patients are transplanted with a MELD score >30 [6,8].

Greater surgical expertise, better selection of patients, improved post-liver transplantation management of complications [1], and introduction of potent immunosuppressants, antibiotics and antiviral drugs are the key reasons for improvement in the survival rates [9]. Calcineurin inhibitors (CNIs) are the mainstay of immunosuppressive therapy in liver transplantation. However, post-transplant long-term use of CNIs is associated with a higher risk of chronic renal failure in liver transplant recipients [10,11] and in renal transplantation recipients [12]. Among the liver transplant recipients, approximately 20% of patients develop chronic renal failure (glomerular filtration rate of 29 mL/min/1.73 m^2^) by 5 years [10], leading to increased mortality [13,14]. Prolonged CNI exposure is also associated with neurotoxicity, *de novo* malignancies, recurrence of hepatitis C viral (HCV) infection and hepatocellular carcinoma (HCC) [15], and an increased risk of metabolic complications [11]. Therefore, it is important to identify alternate immunosuppressive regimens that: (1) maintain efficacy similar to CNI and optimize renal function while reducing CNI exposure and thus related nephrotoxicity; (2) minimize CNI-associated adverse events; and (3) reduce the post-transplant recurrence of HCV and HCC and occurrence of *de novo* malignancies [15].

### Eliminating/reducing calcineurin inhibitor exposure: mammalian target of rapamycin inhibitors

Mammalian target of rapamycin (mTOR) inhibitor (everolimus, sirolimus)-based CNI reduction or elimination is being practiced to overcome drug-induced adverse events. mTOR inhibitor-enabled reduced CNI exposure offers renal benefits without affecting efficacy in low-to-moderate risk *de novo* kidney transplant recipients [12]. Emerging data suggest that mTOR inhibitors offer antiviral benefits against BK virus, human papilloma virus, cytomegalovirus (CMV), human herpes virus 8 and several other herpes viruses [16]. Early initiation of mTOR inhibitor-based immunosuppression is more effective in reducing the risk of CMV infection and disease in solid organ transplant recipients [17]. Furthermore, a probable negative impact of mTOR inhibitors in post-operative surgical complications [15,18] was contradicted by findings from a single-center study in six liver transplant recipients, indicating that the rate of complications after major surgery is similar in patients receiving mTOR inhibitors to those not receiving mTOR inhibitors [19].

### Everolimus in liver transplantation

Studies in *de novo* and maintenance liver transplant recipients demonstrated that everolimus facilitates CNI reduction/elimination without compromising efficacy (Table 1). Using an appropriate dose and switching to everolimus within 3 months of transplantation optimizes renal function and minimizes CNI-induced adverse events with comparable efficacy [20-32]. Other potential benefits of mTOR inhibitors related to HCV-related fibrosis, metabolic syndrome, and neurotoxicity have long-term implications for liver transplant recipients [15].

**Table 1:** **Everolimus in liver transplantation**

**Study**	**Participants**	**Study design and duration**	**EVR dosing**	**Efficacy**	**Renal function**	**Safety**
**Early conversion (≤3 months after transplantation)**						
Levy *et al*. [20]	N = 119 recipients	Prospective, randomized; 36 months	1, 2, or 4 mg/day	32.1, 26.7, and 25.8 for EVR 1, 2, and 4 mg/day at 1 year after immunosuppression was initiated; 39.3, 30.0, and 29.0 for EVR 1, 2, and 4 mg/day at 3 years after immunosuppression was initiated	Change in CrCl (mL/min) from baseline at 1 year post-conversion: EVR 1, 2, and 4 mg/day vs placebo: −20.2, −43.0, and −36.9 vs −36.9	• CMV disease: 3.3, 3.6, 6.7, and 9.7 (placebo vs EVR 1, 2, and 4 mg/day, respectively, *P* = NS for all comparisons)
	• n = 28 (EVR 1.0 mg) + CNI					
	• n = 30 (EVR 2.0 mg) + CNI					
	• n =31 (EVR 4.0 mg) + CNI					
	• n =30 (placebo) + CNI					
						• Thrombocytopenia: 10.0, 14.3, 20.0, and 19.4
						• Leukopenia: 0, 14.3, 6.7, and 6.5
Masetti *et al*. [21]	N = 78 recipients	Prospective, randomized; 12 months	Initial dose: 2.0 mg/day, C0 → 6–10 ng/mL post- CsA withdrawal: C0 → 8–12 ng/mL until Month 6, and C0 → 6–10 ng/mL thereafter	5.7 vs 7.7 at 40–87 days vs at 41–240 days after transplant (NS)	Change in GFR (mL/min/1.73 m^2^) 1 year after conversion: EVR vs CsA: +5.9 vs −14.8 (*P* < 0.001)	EVR vs CsA
						• Inferior limb edema: 9.6 vs 0
	• n = 52 (EVR)					
	• n = 26 (CsA)					• Incisional hernia: 46.1 vs 26.9
						• Biliary complications (stenosis/leak): 21.1 vs 30.8
						• Infections: 46.1 vs 46.1
						• CMV: 19.2 vs 23.1
						(*P =* NS for all comparisons)
Fischer *et al*. [22]	N = 203 recipients on CNI without corticosteroids	Prospective, randomized; 12 months	Initial dose of 1.5 mg b.i.d.; target C0 → 5–12 ng/mL in patients on treatment with TAC; C0 → 8–12 ng/mL in patients on treatment with CsA	EVR vs CNI control at 11 months: 17.7 vs 15.3	EVR vs CNI: change in GFR (mL/min/1.73 m^2^)11 months after conversion from baseline (MDRD): 2.0 ± 23.2 vs −2.8 ± 23.1; LS mean difference ± SE: −7.778 ± 3.338 (*P* = 0.021)^a^	EVR vs CNI
						• Wound complications: 2 vs 3.9
	• n = 101 (EVR)					• Incisional hernia: 11.9 vs 9.8
	• n = 102 (CNI continuation)					
						• Wound dehiscence: 0 vs 1
						• Wound hemorrhage: 1 vs 0
						• Infections and infestations: 73.3 vs 59.8
						• Anemia: 18.8 vs 10.8
						• Leukopenia: 20.8 vs 9.8^b^
						• Thrombocytopenia: 7.9 vs 6.9 (*P* = NS for anemia and thrombocytopenia)
Sterneck *et al*. [23]	N = 81 recipients from Fischer *et al*. [22]	Prospective, randomized; 35 months	Same as Fischer *et al*. [22]	EVR vs CNI control at 35 months: 24.4 vs 15.8 (P = NS)	Change in GFR 35 months after conversion: difference in eGFR between EVR and CNI (CG): −10.5 mL/min (*P* = 0.096) and Nankivell formula: −10.5 mL/min (*P* = 0.015)	EVR vs CNI
						• Peripheral edema: 22.0 vs 5.0 (*P* = 0.048)
						• Neoplasms:17.1 vs 19.8 (*P* = 0.587)
	• n = 41 (EVR ± corticosteroids)					
	• n = 40 (CNI ± corticosteroids)					• Incisional hernia: 24.4 vs 15.0 (*P =* 0.404)
						• Anemia: 4.9 vs 5.0 (*P =* 1.000)
De Simone *et al*. [24]	N = 719 recipients	Prospective, randomized; 12 months	EVR + TAC-WD: initial dose of 1.0 mg b.i.d. ≤24 hours of randomization and C0 3–8 ng/mL until Month 4 post-Tx. Target C0 increased to 6–10 ng/mL. EVR + rTAC: initial dose of 1.0 mg b.i.d. ≤24 hours of randomization and C0 3–8 ng/mL maintained throughout the study. Recruitment to EVR + TAC-WD arm was terminated early.	EVR + rTAC vs TAC-C: 4.1 vs 10.7, *P* = 0.005	Change in GFR (mL/min/1.73 m^2^) 1 year after conversion: adjusted mean difference in eGFR change for EVR + rTAC vs TAC-C: 8.50 ± 2.12; *P* < 0.001)	EVR + rTAC vs TAC-C:
	• n = 245 (EVR + rTAC)					• Edema: 17.6 vs 10.8 (RR 1.63; 95% CI: 1.03, 2.56)
	• n = 231 (EVR + TAC-WD)					
						• Wound complications: 11.0 vs 7.9 (RR 1.40; 95% CI: 0.80, 2.45)
	• n = 243 (TAC-C)					
						• Incisional hernia: 2.9 vs 1.2 (RR 2.30, 95% CI: 0.60, 8.77)
						• Leukopenia: 11.8 vs 5.0 (RR 2.38, 95% CI: 1.24, 4.55)
						• Thrombocytopenia: 5.3 vs 1.7
						• Anemia: 7.8 vs 8.3 (RR 0.93, 95% CI: 0.51, 1.71)
Saliba *et al*. [25]	Same as De Simone *et al*. [24]	Prospective, randomized; 24 months	Same as De Simone *et al*. [24]	6.1 vs 13.3; −7.2% (97.5% CI: −13.5, −0.9; *P =* 0.010). EVR + rTAC vs TAC-C at 24 months)	Change in GFR (mL/min/1.73 m^2^) 24 months after conversion: EVR + rTAC vs TAC-C: mean difference in eGFR change: +6.7 (97.5% CI: +1.9, +11.4; *P* = 0.002)	EVR + rTAC vs TAC-C:
						• Peripheral edema: 22.4 vs 14.9 (*P =* 0.036)
						• Wound complications: 11.0 vs 8.3 (*P =* 0.36)
						• Incisional hernia: 9.8 vs 7.9 (*P =* 0.52)
						• Thrombocytopenia: 8.2 vs 2.9 (*P =* 0.016)
						• Anemia: 9.8 vs 10.3 (*P =* 0.88)
						• CMV: 4.9 vs 5.4 (*P =* 0.84)
						• Viral infection: 18.4 vs 18.2 (*P =* 1.000)
**Late conversion (>3 months after transplantation)**						
Bilbao *et al*. [26]	N = 25 recipients.	Retrospective; mean of 10 ± 9 months	In refractory rejection: initial dose 0.5 mg/12 hours (C0 → 5 ng/mL). For CNI-related adverse events: 0.5 mg once/twice a day. For malignancy: 0.5 mg/day, C0 < 3 ng/mL			• Mucositis: 4
	All converted to EVR					• Sepsis (graft-vs-host disease): 4
Casanovas *et al*. [27]	N = 35 recipients. All converted to EVR	Prospective, single-arm; mean of 134 months	Initial dose 0.25 mg/12 hours for the first 4 days. Target C0 3–5 ng/mL			Anemia, leukopenia, and thrombocytopenia: 11.4
Castroagudin *et al*. [28]	N = 21 recipients (chronic renal dysfunction). All converted to EVR	Prospective, single-arm; median of 19.8 months	0.75 mg b.i.d., C0 → 3–8 ng/mL		Change in GFR (mL/min/1.73 m^2^) 1 year after conversion: +7.65 (*P =* 0.016 vs baseline)	
De Simone *et al*. [29]	N = 40 recipients	Prospective, single-arm 12 months	1.5 mg/day (C0 → 3–8 ng/mL)	tBPAR: 15	Change in CrCl (mL/min) 1-year post conversion: 4.03±12.6 (-10.6-52.5)	• Oral ulcers/stomatitis: 22.5
						• Lower urinary tract infection: 5
						• Pruritis and acne:7.5 each
De Simone *et al*. [30]	N = 145 recipients	Prospective, randomized; 12 months	Initial dose of 3 mg/day b.i.d on day 1. After week 2: EVR C0 → 3–8 ng/mL with concomitant CNI or C0 → 6–12 ng/mL if CNI was eliminated	EVR vs CNI: 4.2 vs 1.4	Change in CrCl (mL/min) 6 months post-conversion: EVR: +1.0; controls: +2.3 (NS)	EVR vs CNI
						• Mouth ulcers: 26.4 vs 0.0 (*P* < 0.01)
	• n = 72 (EVR therapy with CNI reduction or discontinuation)					
						• Infections: 31.9 vs 21.9 (15.3 vs 1.4 suspected to be drug-related)
	• n = 73 (CNI continuation)					
						• Rash/dry skin/eczema: 6.9 vs 0.0 (*P =* 0.028)
						• Leukopenia: 12.5 vs 5.5
						• Thrombocytopenia: 5.6 vs 1.4
						• Anemia: 9.7 vs 4.1
Saliba *et al*. [31]	N = 240 maintenance recipients. All received EVR	Retrospective; 12 months	Introduced at mean 2.4 mg/day (Month 1: C0 → 7.3 ng/mL, Month 12 C0 → 8.1 ng/mL) C0 → 8.8 ng/mL at Month 12 in monotherapy cohort	BPAR: 1.6	Change in GFR mL/min/1.73 m^2^; (CG method) 1 year after conversion (overall vs baseline): +4.2 (*P* = 0.007) chronic renal failure (subpopulation vs baseline): +8.6 (*P* = 0.02)	• Edema: 16.3
						• Stomatitis/mouth ulcers: 14.2
						• Bacterial infection:12.5
						• Rash: 18.8
						• Anemia: 12.9
						• Leukopenia: 9.2
						• Thrombocytopenia: 6.3
Vallin *et al*. [32]	N = 94 recipients.	Retrospective; mean 12 ± 7 months	Initial dose of 0.75–1.5 mg b.i.d. C0 adjusted to 3–8 ng/mL	9		• Edema: 7
	All received EVR					• Mucositis: 15
						• Infection: 3
						• Dermatitis: 19

^a^Between-group difference (calculated as CNI group minus everolimus group) at Month 11 after baseline; results based on analysis of covariance model. ^b^Treatment group differences with an exploratory *P* value of ≤0.05. *P* values are included where available. b.i.d., twice daily; BPAR, biopsy-proven acute rejection; C0, trough level; CG, Cockcroft-Gault; CI, confidence interval; CMV, cytomegalovirus; CNI, calcineurin inhibitor; CrCl, creatinine clearance; CsA, cyclosporine A; eGFR, estimated glomerular filtration rate; EVR, everolimus; GFR, glomerular filtration rate; LS, least square; MDRD, modification of diet in renal disease; NS, nonsignificant; RR, relative risk; rTAC, reduced tacrolimus; SE, standard error; TAC, tacrolimus; TAC-C, standard tacrolimus; TAC-WD, tacrolimus withdrawal; Tx, transplantation.

H2304, the registry study for everolimus use in liver transplantation, reported beneficial effects of everolimus [25]. Results from the H2304 study suggested that, despite the beneficial effects of everolimus initiation 30 ± 5 days post-transplantation, incidences of CMV and HCC recurrence were comparable (CMV: 4.9% versus 5.4%, *P* = 0.84; and HCC recurrence: 1.2% versus 1.2%, *P* = 1.0) between the everolimus plus reduced tacrolimus and the standard tacrolimus arms, respectively, at 24 months [25].

Initiation of everolimus earlier than 30 ± 5 days post-transplantation might provide antiviral benefits. Therefore, the Hephaistos study (NCT01551212; Efficacy of Everolimus in Combination With Tacrolimus in Liver Transplant Recipients) aims to establish the beneficial effects of early initiation of everolimus and the impact on the development or the rate of progression of fibrosis in HCV-positive recipients without affecting wound healing. A very low initial immunosuppressant dosage has been used, considering the relevant number of patients in Germany with a MELD score >30, who are in a poor health condition at the time of transplantation.

## Methods/Design: Hephaistos study

### Overview

**Figure 1:**
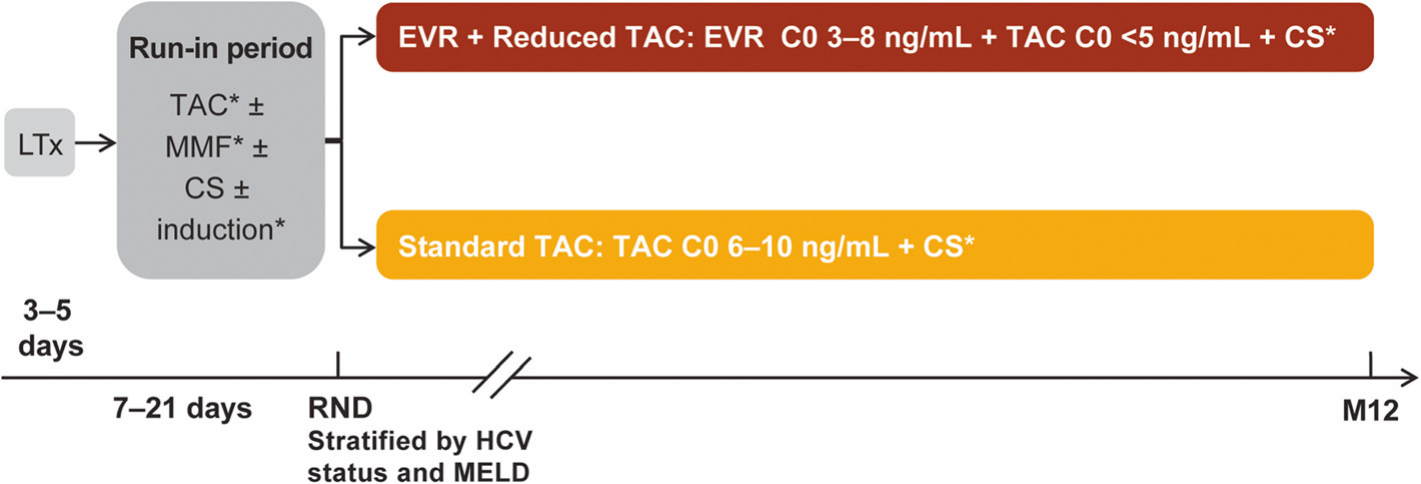
**Study design.** *As per center practice. C0, trough levels; CS, corticosteroids; EVR, everolimus; HCV, hepatitis C virus; LTx, liver transplantation; M, month; MELD, model of end-stage liver disease; MMF, mycophenolate mofetil; RND, randomization; TAC, tacrolimus.

Hephaistos (protocol version 3, 14 June 2013) is an ongoing, 12-month, multicenter, open-label, randomized, controlled study aimed to evaluate efficacy, safety, and evolution of renal function of everolimus with reduced tacrolimus in *de novo* liver transplant recipients. Patients undergoing a successful liver transplantation enter a run-in period between 3 and 5 days post-transplantation. During the run-in period, induction therapy, mycophenolate mofetil, tacrolimus and corticosteroids are initiated at the investigator’s discretion. Between 7 and 21 days post-transplantation, patients are randomized in a 1:1 ratio to receive either: (i) everolimus (trough level (C0) 3–8 ng/mL) with reduced tacrolimus (C0 <5 ng/mL), or (ii) standard tacrolimus (C0 6–10 ng/mL; Figure 1). Everolimus is initiated on the day of randomization and will be monitored throughout the study period (post 5 ± 2 days of everolimus/tacrolimus dose changes).

### Study population

This study plans to enroll approximately 330 *de novo* liver transplant recipients, 165 in each study arm, from 15 transplant centers in Germany. The study population comprises adult patients aged between 18 and 65 years receiving a liver transplant from a deceased or living donor. At the time of randomization, only patients with an acceptable renal function defined as estimated glomerular filtration rate (eGFR) >30 mL/min/1.73 m^2^ assessed by the Modification of Diet in Renal Disease (MDRD)-4 formula [33] (obtained within 5 days before randomization) are eligible to enter the study. Inclusion and exclusion criteria at the time of study entry and randomization are summarized in Table 2. The study was approved by all competent Ethics Committees and regulatory authorities (Additional files 1 and 2). Informed consent is being obtained from all patients enrolled to the study by the investigators.

**Table 2:** **Key inclusion and exclusion criteria for the Hephaistos study**

**Inclusion criteria**	**Exclusion criteria**
1. Patients able to give written informed consent to participate in the study	1. Recipients of multiple solid organ transplants
2. Male or female recipients 18–65 years with full-size liver allograft	2. Patients with renal failure or CKD/ESRD requiring renal replacement therapy for more than 2 weeks prior to transplantation
3. Negative pregnancy test (females of child bearing age)	3. History of malignancy within the past 5 years
	4. HCC that does not fulfill the Milan criteria at the time of transplantation
	5. Patients with a known hypersensitivity to the drugs used in the study or their class, or to any of the excipients
	6. Recipients of ABO incompatible transplant grafts
	7. HIV positive patients
	8. Patients with a current systemic infection or sepsis requiring active use of intravenous antibiotics
At randomization (Day 7–21 post-liver transplantation)	
1. eGFR (MDRD4 formula) >30 mL/min/1.73 m^2^	1. Platelet count <50,000/mm^3^, absolute neutrophil count <1,000/mm^3^, or a white blood cell count <2,000/mm^3^
2. Absence of thrombosis prior to any initiation of treatment with everolimus	2. Hemoglobin <8.0 g/dL
3. Functioning allograft (total bilirubin levels ≤3 times ULN, and AP, AST, and ALT levels ≤5 times ULN)	3. Uncontrolled hypercholesterolemia or hypertriglyceridemia
	4. Proteinuria >1 g/24 hours
	5. Patients with infections requiring active use of intravenous antibiotics

ALT, alanine aminotransferase; AP, alkaline phosphatase; AST, aspartate aminotransferase; CKD, chronic kidney disease; eGFR, estimated glomerular filtration rate; ESRD, end-stage renal disease; HCC, hepatocellular carcinoma; MDRD4, Modification of Diet in Renal Disease-4; ULN, upper limit of normal.

### Study objectives

This study is designed to evaluate the renal function, efficacy and safety of everolimus with reduced tacrolimus in liver transplant recipients. The primary objective of the study is to demonstrate superior eGFR (MDRD-4 formula) with the everolimus plus reduced tacrolimus regimen compared with standard tacrolimus at Month 12. The key secondary objective is to evaluate the incidence of treated biopsy-proven acute rejection, graft loss, or death at Month 12. Other secondary objectives are to evaluate: (i) incidences of components of the composite efficacy endpoint; (ii) renal function by eGFR using various formulae (MDRD-4, Nankivell, Cockcroft-Gault, chronic kidney disease epidemiology collaboration and Hoek formulae); (iii) incidence of proteinuria; (iv) incidence of adverse events and serious adverse events; and (v) incidence and severity of CMV and HCV infections and HCV-related fibrosis. Efficacy and safety objectives of the study are summarized in Table 3. All adverse events and serious adverse events occurring after administration of study treatment will be documented in case report forms and patient medical records for further monitoring.

**Table 3:** **Objectives of the Hephaistos study**

Primary objective	To demonstrate that an immunosuppressive regimen based on everolimus with reduced tacrolimus has superior efficacy compared with tacrolimus alone on eGFR (MDRD-4 formula) at Month 12 in *de novo* liver transplant recipients
Key secondary objective	To evaluate the incidence of a composite of tBPAR, graft loss, or death at Month 12
Other key secondary efficacy objectives	• Incidence of individual and composite efficacy components (tBPAR, graft loss, death, or loss to follow-up) at Months 6 and 12
	• To evaluate treated BPAR by: (1) incidence, (2) time to event, (3) severity, and (4) diagnosis leading to transplantation
	• To evaluate any acute rejection by: (1) incidence, (2) time to event, and (3) severity
Other key renal function-related objectives	• Evolution of renal function (eGFR; MDRD-4) over time
	• Renal function (eGFR by MDRD-4, Nankivell, Cockcroft-Gault, CKD-EPI, and Hoek formulae)
	• To evaluate serum creatinine at various time points
	• To evaluate renal function and change in eGFR from screening, randomization, and Week 2 post-transplantation to Months 6 and 12 in various subgroups
	• To evaluate urinary protein/creatinine ratio and incidence of proteinuria at various time points
Other key safety-related objectives	• Incidence of adverse events/infections/serious adverse events
	• Incidence of treatment-related side effects, such as NODM, evolution of metabolic parameters as subdivisions of serum/plasma lipid panel, neurotoxicity, and hypertension
	• Incidence and reason for premature discontinuation of study medication and premature discontinuation from the study
Key virus (HCV and CMV)- and HCC-related objectives	• Incidence and rate of progression of HCV, HCV-related fibrosis, and HCV viral load
	• Incidence of and response to HCV antiviral treatment
	• Incidence of *de novo* HCC malignancies and rate of recurrence at Month 12
	• Incidence and severity of CMV viral infections

BPAR, biopsy-proven acute rejection; CKD-EPI, chronic kidney disease epidemiology collaboration; CMV, cytomegalovirus; eGFR, estimated glomerular filtration rate; EVR, everolimus; HCC, hepatocellular carcinoma; HCV, hepatitis C virus; MDRD, modification of diet in renal disease; NODM, new onset diabetes mellitus; rTAC, reduced tacrolimus; tBPAR, treated biopsy proven acute rejection.

### Randomization and immunosuppression

*De novo* liver transplant recipients are randomized 7–21 days post-transplantation to receive everolimus plus reduced tacrolimus or standard tacrolimus. All eligible patients are randomized using a validated system that automates the random assignment of treatment arms in the specified ratio. Randomization is stratified by HCV status (positive/negative) and the laboratory MELD score below/equal/above 30 at transplantation.

Immunosuppression up to the point of randomization comprises induction therapy, mycophenolate mofetil, tacrolimus, and corticosteroids administered according to local practice. Mycophenolic acid is discontinued at the time of randomization as per local practice. Patients in the everolimus plus reduced tacrolimus group are administered everolimus on the day of randomization at a dose of 1.0 mg b.i.d The target therapeutic range for everolimus is maintained at 3–8 ng/mL and dose adjustments will be monitored 5 ± 2 days post-randomization. Tacrolimus dose is adjusted to achieve a C0 <5 ng/mL after everolimus C0 is achieved. In the standard tacrolimus group, the target tacrolimus C0 is 6–10 ng/mL. Patients in both the groups receive corticosteroids at the investigator’s discretion. The treatment regimens will be continued until Month 12 post-randomization. Dose adjustments and interruptions are allowed only in patients who are unable to tolerate the protocol-specified dosing scheme. Everolimus dose will be: (i) interrupted in severe hematological adverse events until the condition is resolved; and (ii) permanently discontinued if a hemolytic uremic syndrome occurs. Tacrolimus dose will be adjusted if the whole blood levels are outside the target range and reduced in case of tacrolimus toxicity. Follow-up medical care is provided to all patients who prematurely discontinue from the study as per center practice.

Rejection episodes are treated as per local practice or at the investigator’s discretion. Other concomitant medications such as prophylactic treatment of CMV with valganciclovir, ganciclovir, CMV hyperimmune globulin, acyclovir or valacyclovir; *Pneumocystis carinii* with Bactrim® or equivalent (trimethoprim/sulfamethoxazole), oral candida with nystatin suspension and treatment of hepatitis B and C virus prophylaxis are permitted as per local practice.

### Statistical analysis

This study tests the null hypothesis that there is zero treatment difference in the mean eGFR between the everolimus plus reduced tacrolimus and standard tacrolimus groups at Month 12 after baseline. The null hypothesis is tested with analysis of covariance with treatment, center, HCV class (positive/negative), and MELD (≤30 vs >30) as factors, and eGFR at Visit 1 (baseline) as a covariate. An alternative hypothesis assumes that the difference between the two arms is 7.0 mL/min/1.73 m^2^. A sample size of 105 in each group will have 80% power to detect a difference of 7.0 mL/min/1.73 m^2^ in the mean eGFR, assuming that the common standard deviation is 18.0 mL/min/1.73 m^2^ with a 5% two-sided significance level. Summary statistics of demographic and baseline characteristics, efficacy observations and measurements, safety observations and measurements, and pharmacokinetic measurements will be presented by treatment group. Categorical variables will be summarized by absolute and relative frequencies, and continuous variables will be summarized by descriptive statistics. Time-to-event data including rates of affected patients will be assessed by Kaplan-Meier statistics. Group comparisons will be performed using appropriate two-sided statistical tests. Physiological, laboratory and clinical assessments will be carried out as per plan (Additional file 3). Full analysis set will be used for the analysis of data.

Study conduct, analysis of study outcomes and documentation of the study results will be in accordance with the Declaration of Helsinki [34], Good Clinical Practice [35], and the local legal and regulatory requirements. Trial centers will be randomly audited by funding authorities. All data management activities will be carried out by a data monitoring committee according to the current standard operating procedures of Novartis Pharma GmbH, Nürnberg, Germany. The data monitoring committee has the right and duty to recommend study closure if necessary.

### Sub-studies

The Hephaistos study will also include five sub-studies to evaluate:

1. Importance of non-human leukocyte antigen (HLA) antibodies targeting G-protein coupled receptor in liver transplant pathologies. This sub-study will determine: (i) the anti-AT_1_-receptor and anti-ET_A_-receptor antibodies status; (ii) correlation with immunologic and non-immunologic events; (iii) the correlation with histopathologic findings, if a biopsy is available; and (iv) functional outcome.
2. Cytochrome P450 (CYP450)-dependent vasoactive eicosanoids in serum and urine as a marker and mediator of nephrotoxicity after liver transplantation. This sub-study will determine: (i) CYP450-eicosanoid profile in plasma and urine and correlation with kidney and liver function as well as histopathologic findings if a biopsy is available; and (ii) genetic polymorphisms in CYP-isoforms (CYP3A4 and CYP3A5), drug transporters (ABCB1), and catechol-o-methyltransferase in donor and recipient, and correlation with renal and liver transplant outcomes.
3. Immunomodulatory effect of everolimus on natural killer cell subsets and plasma cytokine, chemokine and growth factor levels in patients after liver transplantation. The objectives of this sub-study are: (i) to determine alterations in the major natural killer cell subsets defined by CD56, CD16, and CD6 surface expression as well as in CD56 and CD6 expression on CD4+ or CD8+ T cells, respectively, in whole blood of patients before and after liver transplantation; (ii) to determine the individual course of the Th1, Th2, and Th17 response in combination with the chemokine and growth factor response in the plasma of liver transplant recipients; and (iii) to determine the correlation of these immune markers with the type of immunosuppression in both the treatment groups and with the clinical course after transplantation (rejection episodes).
4. Impact of everolimus on the development of alloimmunity and tolerance. The objectives of this sub-study are: (i) to determine the *de novo* HLA antibody development and its correlation with humoral rejection (for example, biliary alteration); (ii) to determine the changes in the T-cell activation marker sCD30, tolerance marker HLA-G, and regulatory antibody IgA-anti-Fab; and (iii) to determine the changes in regulatory T- and B-cell populations under everolimus and CNI minimization during the first year after transplantation. In addition, the study team plans to investigate the association between the immunological changes observed during the first year and outcome during the first 2 years after transplantation.
5. Immunomodulatory effect of everolimus on regulatory and innate lymphocyte populations and on CMV-specific T-cell immunity. The objective of this sub-study is to test if the immunosuppressive drug regimen differentially affects individual immunocompetence towards CMV, CMV-specific T-cell frequencies, phenotype, and functionality. The regulatory T-cells will be analyzed in parallel with CMV load analyses.

Blood samples will be drawn at screening, baseline, and Days 90 and 360 for the sub-studies and processed to obtain serum and/or plasma. For evaluating the genetic polymorphisms, blood samples from recipients and donors are collected in EDTA at screening. Liver biopsy is preserved if donor blood is not available. The protocol was amended in June 2013 to incorporate the details of the sub-studies.

## Discussion

This study aims to demonstrate superior renal function, comparable efficacy, and safety in patients receiving everolimus plus reduced tacrolimus versus the standard tacrolimus regimens. This study also evaluates the antiviral benefit by initiation of everolimus as early as Day 7. This study allows very low initial immunosuppression because of a relevant number of high-MELD patients in Germany with a poor initial condition.

## Trial status

Hephaistos is an ongoing study recruiting *de novo* liver transplant recipients at 15 centers across Germany. Currently 359 patients are screened and, of these, 156 patients are randomized (78 in each arm). At baseline, demographic characteristics such as mean age (54.04 versus 54.21 years), male patients (77.8% versus 62.5%), Caucasian patients (100% versus 95.8%) and mean body mass index (26.01 versus 26.81 kg/m^2^) were similar in the everolimus with reduced tacrolimus and standard tacrolimus regimens, respectively. All investigators are involved in final data interpretation and analysis. All trial results will be disclosed to the health authorities with subsequent publication in NIM and journals.

## List of study sites

Universitaetsklinikum Hamburg-Eppendorf Hepatobiliare Chirurgie; Medizinische Hochschule Hannover; Charite Berlin Campus Virchow-Klinikum Allg.-,Visceral-,Transpl.-Chir; Universitatsklinikum Regensburg Chirurgie; Klinikum der Universitaet Heidelberg; Universitaetsklinikum Schleswig-Holstein Campus Kiel Allgemein- und Thoraxchirurgie; Universitatsklinikum Leipzig AoR Chirurgische Klinik II; Klinikum der Johannes-Gutenberg Universitaet; Universitatsklinikum Essen gGmbH Lebertransplantationsambulanz; Klinikum Grosshadern Muenchen Chirurgische Klinik Poliklinik; Universitaetsklinikum Frankfurt Chirurgie; Universitatsklinikum Tubingen Chirurgie; Universitatsklinikum Aachen Allgemein, Viszeral, Transpl.; Universitatsklinikum Erlangen Chirurgische Klinik; Universitatsklinikum Bonn Allg./Viszeral/Thorax/Gefass.

## Additional files

Additional file 1:
**List of ethics committees [in German].**
Additional file 2:
**List of ethics committees [in English].**
Additional file 3:
**Assessment schedule.**

## Abbreviations

ALT: alanine aminotransferase
AP: alkaline phosphatase
AST: aspartate aminotransferase
BPAR: biopsy-proven acute rejection
C0: trough level
CG: Cockcroft-Gault
CKD-EPI: chronic kidney disease epidemiology collaboration
CMV: cyto- megalovirus
CNI: calcineurin inhibitor
CsA: cyclosporine
CYP450: cytochrome P450
eGFR: estimated glomerular filtration rate
ESRD: end stage renal disease
EVR: everolimus
HCC: hepatocellular carcinoma
HCV: hepatitis C virus
HIV: human immunodeficiency virus
HLA: human leukocyte antigen
MDRD: Modification of Diet in Renal Disease
MELD: model of end-stage liver disease
MMF: mycophenolate mofetil
mTOR: mammalian target of rapamycin
NODM: new onset diabetes mellitus
TAC: tacrolimus
tBPAR: treated biopsy-proven acute rejection
ULN: upper limit of normal
