# Supplementary material for: Evaluating the efficacy, safety and evolution of renal function with early initiation of everolimus-facilitated tacrolimus reduction in de novo liver transplant recipients: Study protocol for a randomized controlled trial
Source: Trials. 2015 Mar 26;16:118. doi: 10.1186/s13063-015-0626-0 (PMC4384314; doi:10.1186/s13063-015-0626-0)
Supplement: Additional file 1: — List of ethics committees [in German]. [file 13063_2015_626_MOESM1_ESM.pdf]

Liste Ethik-Kommissionen

Federführende Ethik-Kommission

Ärztekammer Hamburg
Ethik-Kommission
Humboldtstr. 67a
22083 Hamburg
Tel. 040-202299240
Fax 040-202299410

Beteiligte Ethik-Kommission

Ethik-Kommission an der Medizinischen Fakultät der RWTH Aachen
Pauwelsstr. 30
52074 Aachen
Tel. 0241-8089963
Fax 0241-8082012

Landesamt für Gesundheit und Soziales
Ethik-Kommission des Landes Berlin
Fehrbelliner Platz 1
10707 Berlin
Tel. 030-90229 1226
Fax 030-90283383

Rheinische Friedrich-Wilhelms-Universität
Ethik-Kommission
Medizinische Fakultät Bonn
Biomedizinisches Zentrum
Sigmund-Freud-Str. 25
53105 Bonn
Tel. 0228-287 51 931
Fax 0228-287 51 932

Friedrich-Alexander-Universität Erlangen-Nürnberg
Medizinische Fakultät
Ethik-Kommission
Krankenhausstr. 12
91054 Erlangen
Tel. 09131-85 22210
Fax 09131-85 26021

Liste Ethik-Kommissionen

Universitätsklinikum Essen
Medizinische Fakultät der Universität Duisburg-Essen
Ethik-Kommission
Robert-Koch-Straße 9-11
45147 Essen
Tel. 0201-723 3637
Fax 0201-723 5837

Ethik-Kommission des Fachbereichs Medizin der
Johann Wolfgang Goethe-Universität
Universitätsklinikum
Theodor-Stern-Kai 7
60590 Frankfurt am Main
Tel. 069-63014597
Fax 069-630183434

Ethik-Kommission der Medizinischen Hochschule
Hannover
Carl-Neuberg-Str. 1
30625 Hannover
Tel. 0511-5329229
Fax 0511-5325423

Ethik-Kommission der Medizinischen Fakultät
Heidelberg
Alte Glockengießerei 11/1
69115 Heidelberg
Tel. 06221-338220
Fax 06221-3382222

Ethik-Kommission der Medizinischen Fakultät der
Christian-Albrechts-Universität zu Kiel
Schwanenweg 20
24105 Kiel
Tel. 0431-5971809
Fax 0431-5975333

Liste Ethik-Kommissionen

Ethik-Kommission an der Medizinischen Fakultät der
Universität Leipzig
Haus: Karl-Sudhoff-Institut
Käthe-Kollwitz-Str. 82
04109 Leipzig
Tel. 0341-97 15490
Fax 0341-97 15499

Ludwig-Maximilians-Universität München
Klinikum der Universität
Ethik-Kommission
Pettenkoferstr. 8a
80336 München
Tel. 089-5160 5191
Fax 089-5160 5192

Technische Universität München
Fakultät für Medizin
Ethikkommission
Ismaninger Str. 22
81675 München
Tel. 089-41404371
Fax 089-41404199

Ethikkommission
an der Universität Regensburg
Klinikum der Universität Regensburg
Franz-Josef-Strauß-Allee 11
93053 Regensburg
Tel. 0941-944 5380
Fax 0941-944 5388

Landesärztekammer Rheinland-Pfalz
Ethik-Kommission
Deutschhausplatz 3
55116 Mainz
Tel. 06131-2882263
Fax 06131-2882266

Liste Ethik-Kommissionen

Ethik-Kommission
an der Medizinischen Fakultät der
Eberhard-Karls-Universität und
am Universitätsklinikum Tübingen
Gartenstraße 47
72074 Tübingen
Tel. 07071-2977661
Fax 07071-295965
