# Supplementary material for: Evaluating the efficacy, safety and evolution of renal function with early initiation of everolimus-facilitated tacrolimus reduction in de novo liver transplant recipients: Study protocol for a randomized controlled trial
Source: Trials. 2015 Mar 26;16:118. doi: 10.1186/s13063-015-0626-0 (PMC4384314; doi:10.1186/s13063-015-0626-0)
Supplement: Additional file 2: — List of ethics committees [in English]. [file 13063_2015_626_MOESM2_ESM.pdf]

List of ethics committees

Leading Ethics Committee

Hamburg Medical Association Ethics Committee
Humboldtstr. 67a
22083 Hamburg
Tel. 040-202299240
Fax 040-202299410

Participating Ethics Committee

Ethics Committee of the Medical Faculty of the RWTH Aachen
Pauwelsstr. 30
52074 Aachen
Tel. 0241-8089963
Fax 0241-8082012

State Department of Health and Social
Ethics Commission of the State of Berlin
Fehrbellinerplatz 1
10707 Berlin
Tel. 030-90229 1226
Fax 030-90283383

Rheinische Friedrich-Wilhelms-University
Ethics Committee
Faculty of Medicine Biomedical Center Bonn
Sigmund-Freud-Str. 25
53105 Bonn
Tel. 0228-287 51931
Fax 0228-287 51932

Friedrich-Alexander-University of Erlangen-Nuremberg
Faculty of Medicine Ethics Committee
Krankenhausstr. 12
91054 Erlangen
Tel. 09131-85 22210
Fax 09131-85 26021

List of ethics committees

University Hospital Essen
Faculty of Medicine, University of Duisburg-Essen
Ethics Committee
Robert-Koch-Straße 9-11
45147 Essen
Tel. 0201-723 3637
Fax 0201-723 5837

Ethics Committee of the Faculty of Medicine
at the Johann Wolfgang Goethe University,
University Hospital
Theodor-Stern-Kai 7
60590 Frankfurt am Main
Tel. 069-63014597
Fax 069-630183434

Ethics Committee of the Medical School
Hanover
Carl-Neuberg-Str. 1
30625 Hannover
Tel. 0511-5329229
Fax 0511-5325423

Ethics Committee of the Medical Faculty
Heidelberg
Old Bell Foundry 11/1
69115 Heidelberg
Tel. 06221-338220
Fax 06221-3382222

Ethics Committee of the Medical Faculty of the
Christian-Albrechts-University of Kiel
Schwanenweg 20
24105 Kiel
Tel. 0431-5971809
Fax 0431-5975333

List of ethics committees

Ethics Committee of the Medical Faculty of the
University of Leipzig
House: Karl Sudhoff Institute
Käthe Kollwitz Str. 82
04109 Leipzig
Tel. 15490 0341-97
Fax 15499 0341-97

Ludwig-Maximilians-University of Munich
University Hospital
Ethics Committee
Pettenkoferstr. 8a
80336 Munich
Tel. 089-5160 5191
Fax 089-5160 5192

Technical University of Munich
Faculty of Medicine
Ethics Committee
Ismaninger Str. 22
81675 Munich
Tel. 089-41404371
Fax 089-41404199

Ethics Committee
at the University of Regensburg
Regensburg University Hospital
Franz-Josef-Strauss-Allee 11
93053 Regensburg
Tel. 0941-944 5380
Fax 0941-944 5388

State Medical Association Rhineland-Palatinate
Ethics Committee
German court house 3
55116 Mainz
Tel. 06131-2882263
Fax 06131-2882266

List of ethics committees

Ethics Committee
at the Medical Faculty of the
Eberhard-Karls-University and
at the University Hospital of Tübingen
Garden Road 47
72074 Tübingen
Tel. 07071-2977661
Fax 07071-295965
