# Supplementary material for: Evaluating the efficacy, safety and evolution of renal function with early initiation of everolimus-facilitated tacrolimus reduction in de novo liver transplant recipients: Study protocol for a randomized controlled trial
Source: Trials. 2015 Mar 26;16:118. doi: 10.1186/s13063-015-0626-0 (PMC4384314; doi:10.1186/s13063-015-0626-0)
Supplement: Additional file 3: — Assessment schedule. [file 13063_2015_626_MOESM3_ESM.pdf]

Assessment Schedule

	STUDY PERIOD									
Phase	Screening[1]	Baseline[2]	Treatment phase						End of treatment[3]	End of study
Visit	1	2	3	4	5	6	7	8	9a	9
Month					1	3	6	9		12
ENROLLMENT										
Informed consent	X									
Eligibility screen	X	X								
Transplantation information	X									
Randomization		X								
INTERVENTIONS										
Everolimus C0 level			X	X	X	X	X	X	X	X
Tacrolimus C0 level			X	X	X	X	X	X	X	X
ASSESSMENTS										
Demography	X	+++								
General medical history	X									
Physical examination	X	X	X	X	X	X	X	X	X	X
Vital signs	X	X	X	X	X	X	X	X	X	X
Laboratory test: Hemat./Biochemistry		X	X	X	X	X	X	X	X	X
GFR		X	X	X	X	X	X	X	X	X
Urinalysis		X			X	X	X	X	X	X
Viral Serology	X									
CMV monitoring	X	X			X	X	X	X	X	X
HCV monitoring	X	X[4]			X[4]	X[4]	X[4]	X[4]	X[4]	X[4]
Pregnancy test[5] (β-HCG)	X	X								X
Liver Doppler ultrasound[6]		X								
Rejection episodes										
Biopsies										X
Adverse events/infections	X	As necessary								X
Serious adverse events										X
Concomitant therapy										

1. Day 21–day of transplantation.
2. Visit 2 (baseline) has to be performed prior to randomization, 7-21 days after Tx.
3. End of treatment visit in case of premature study discontinuation.
4. Only in HCV positive patients.
5. Only in women of childbearing potential.
6. Liver Doppler ultrasound of the hepatic artery, hepatic vein, portal vein, and inferior vena cava will be carried out up to 5 days prior to randomization.
